# Supplementary material for: The inositol pyrophosphate 5-InsP7 regulates mitochondrial polyphosphate synthesis and bioenergetic function
Source: J Biol Chem. 2026 Mar 31;302(5):111413. doi: 10.1016/j.jbc.2026.111413 (PMC13129382; doi:10.1016/j.jbc.2026.111413)
Supplement: Supporting Figures [file mmc1.pdf]

Supporting information for

The inositol pyrophosphate 5-InsP_7 regulates mitochondrial polyphosphate synthesis and bioenergetic function

Jayashree S. Ladke[1,2], Azmi Khan[1], Anshit Singh[3], Henning J. Jessen[4], Ullas Kolthur-Seetharam[3,5], Manish Jaiswal[6], and Rashna Bhandari[1,*]

[1]Laboratory of Cell Signalling, BRIC-Centre for DNA Fingerprinting and Diagnostics, Hyderabad 500039, India.
[2]Graduate Studies, Regional Centre for Biotechnology, Faridabad, Haryana 121001, India.
[3]Department of Biological Sciences, Tata Institute of Fundamental Research, Mumbai, Maharashtra 400005, India
[4]Institute of Organic Chemistry, University of Freiburg, Albertstr. 21, 79104, Freiburg, Germany
[5]Laboratory of Epigenetics and Ageing, BRIC-Centre for DNA Fingerprinting and Diagnostics, Hyderabad 500039, India.
[6]Tata Institute of Fundamental Research- Hyderabad (TIFR-H), Hyderabad, Telangana 500046, India

This PDF file includes:

Figure and figure legends for supporting information

Figure S1

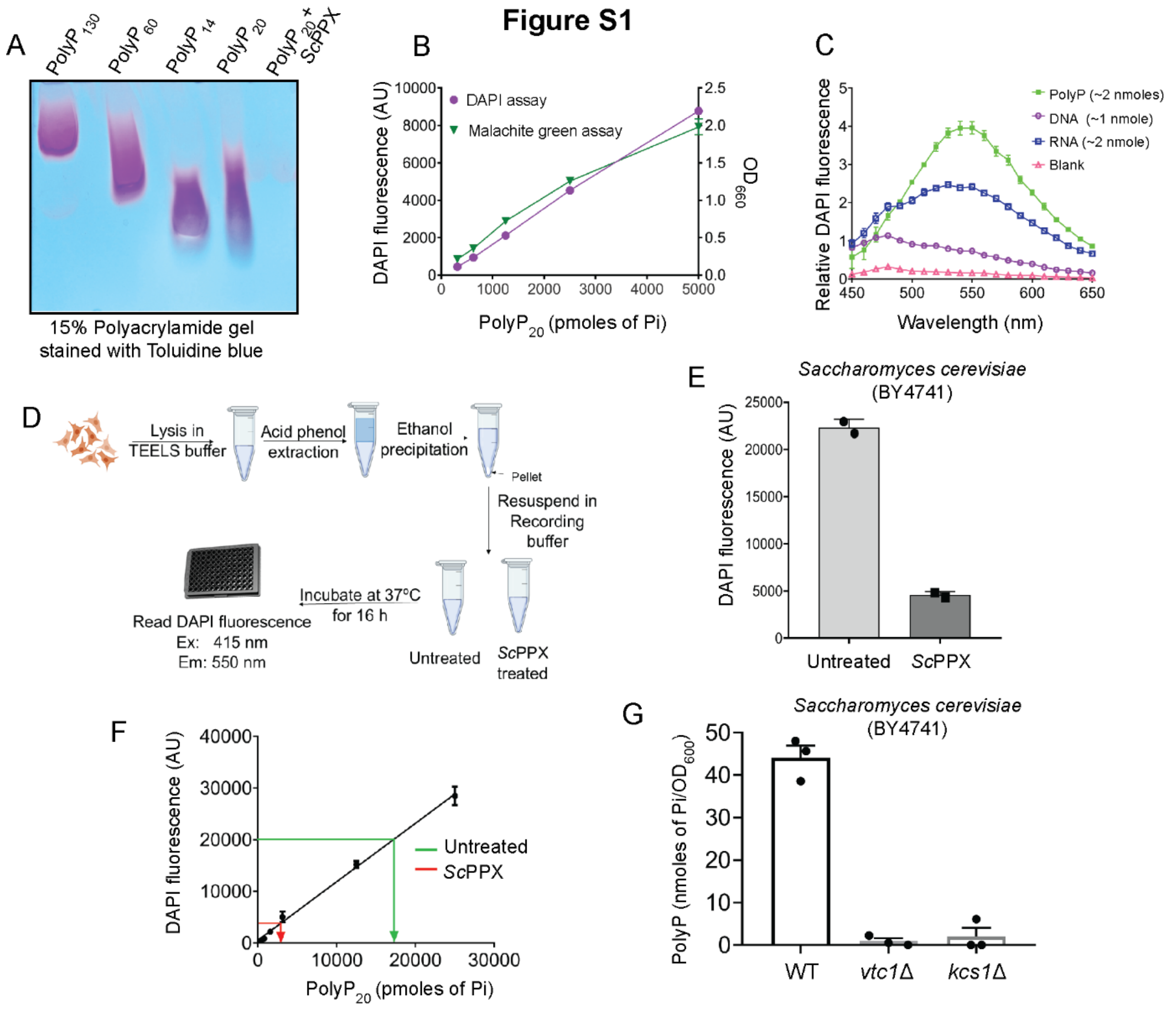

Figure S1. PolyP quantification by DAPI. (A) 15% Tris-borate-EDTA (TBE) polyacrylamide gel stained with toluidine blue representing polyP standards (polyP$_{130}$, polyP$_{60}$, and polyP$_{14}$) from RegeneTiss along with untreated or *Sc*PPX-treated sodium hexametaphosphate, estimating the chain length of the latter to be approximately 20 Pi units (polyP$_{20}$), and demonstrating its linear nature. **(B)** Standard curve for polyP$_{20}$ quantification using Malachite green assay (right Y-axis) and DAPI fluorescence (left Y-axis). Malachite green absorbance (OD: 660 nm) and DAPI fluorescence intensity (Ex: 415 nm, Em: 550 nm) were measured in the presence of increasing amount of polyP$_{20}$ (range: 156 pmoles to 5000 pmoles of Pi). **(C)** Fluorescence emission spectra of DAPI fluorescence obtained upon excitation at 415 nm, with emission measured between 450 nm and 650 nm. Distinct emission profiles were observed for DAPI bound to DNA (purple), RNA (blue), and polyP$_{20}$ (green), compared with blank (pink). Emission maximum was observed at 480 nm for DAPI-DNA and 550 nm for DAPI-RNA and DAPI-polyP$_{20}$. **(D)** Schematic of protocol for polyP extraction and purification, followed by *Sc*PPX treatment and detection by DAPI. **(E)** Quantification of polyP extracted from *S. cerevisiae* (strain BY4741). Bar graph represents DAPI fluorescence intensity for untreated and *Sc*PPX-treated polyP extracts (Ex 415 nm, Em 550 nm). **(F)** Standard curve for polyP$_{20}$ with relative DAPI fluorescence intensities representing the interpolation of the DAPI fluorescence values from bar graph in (E). **(G)** Bar graph representing polyP levels in WT, *vtc1*Δ and *kcs1*Δ *S. cerevisiae* (strain BY4741) normalised to 1 OD culture.

Figure S2

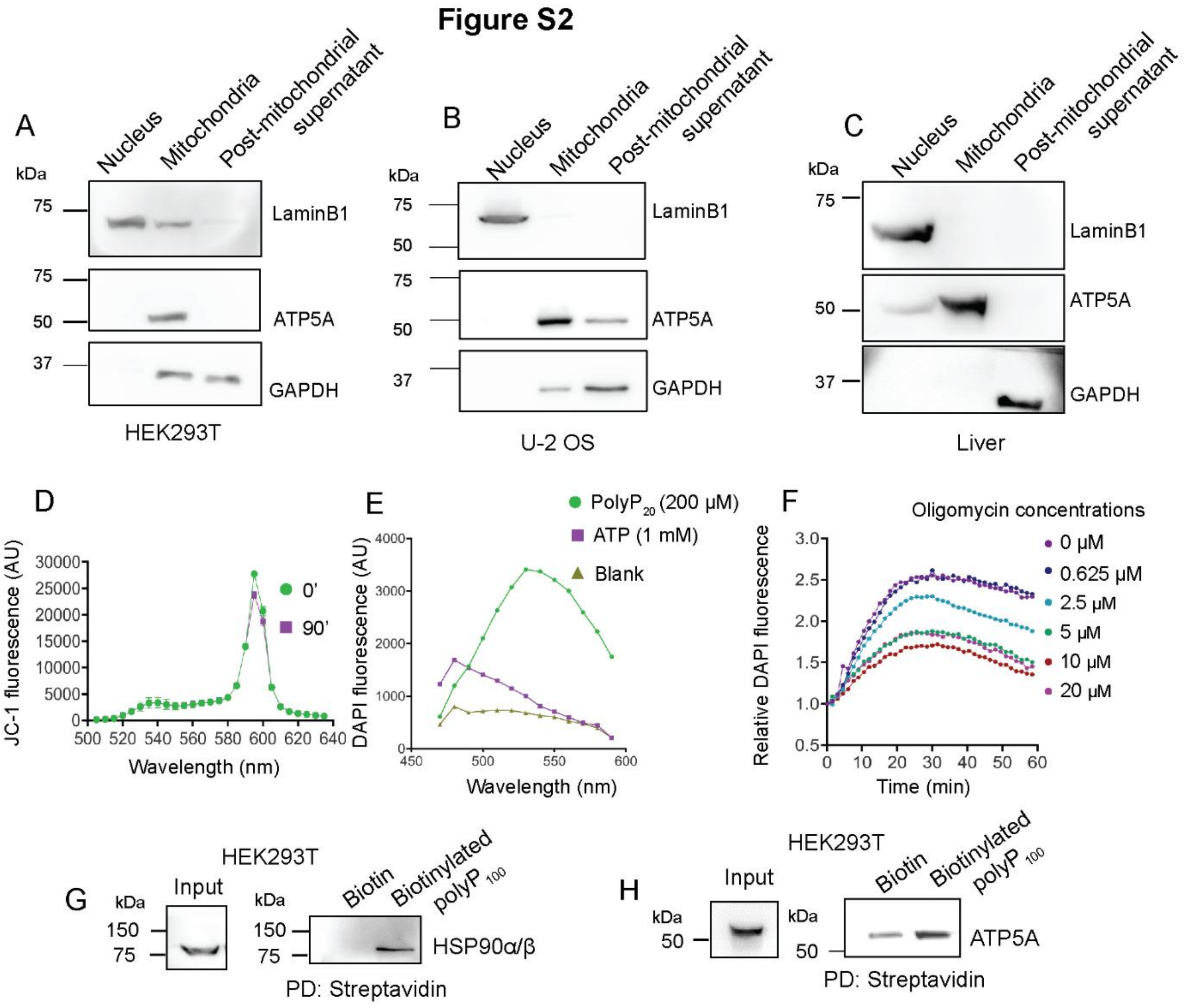

Figure S2. (A-C) Representative immunoblots examining subcellular fractionation of HEK293T (A), U-2 OS cells (B) and mouse liver (C). The nuclear, mitochondrial, and post-mitochondrial fractions were marked by the enrichment of Lamin B1, ATP5A, and GAPDH, respectively. **(D)** JC-1 dye fluorescence spectra of mitochondria isolated from mouse liver incubated in KCl buffer containing glutamate (5 mM), malate (5 mM), and succinate (5 mM), with 0.5 µM JC-1 dye. Spectra were recorded at 0 min (magenta) and 90 min (green) post-incubation. Samples were excited at 488 nm, and emission was recorded from 500 to 640 nm in 10 nm intervals. **(E)** DAPI emission spectra for polyP (200 µM) and ATP (1 mM) upon excitation at 415 nm. **(F)** Line graph depicting the fold change in DAPI fluorescence as a measure of polyP synthesis in isolated mitochondria incubated with glutamate (5 mM), malate (5 mM), succinate (10 mM), Pi (5 mM), and ATP (1 mM) under varying concentrations of oligomycin. **(G-H)** Co-precipitation of endogenous HSP90α/β (G) and ATP5A (H) with biotinylated $polyP_{100}$. HEK293T lysate was incubated with either biotin or biotinylated $polyP_{100}$ immobilized on streptavidin beads and probed to detect endogenous HSP90 α/β or ATP5A.

Figure S3

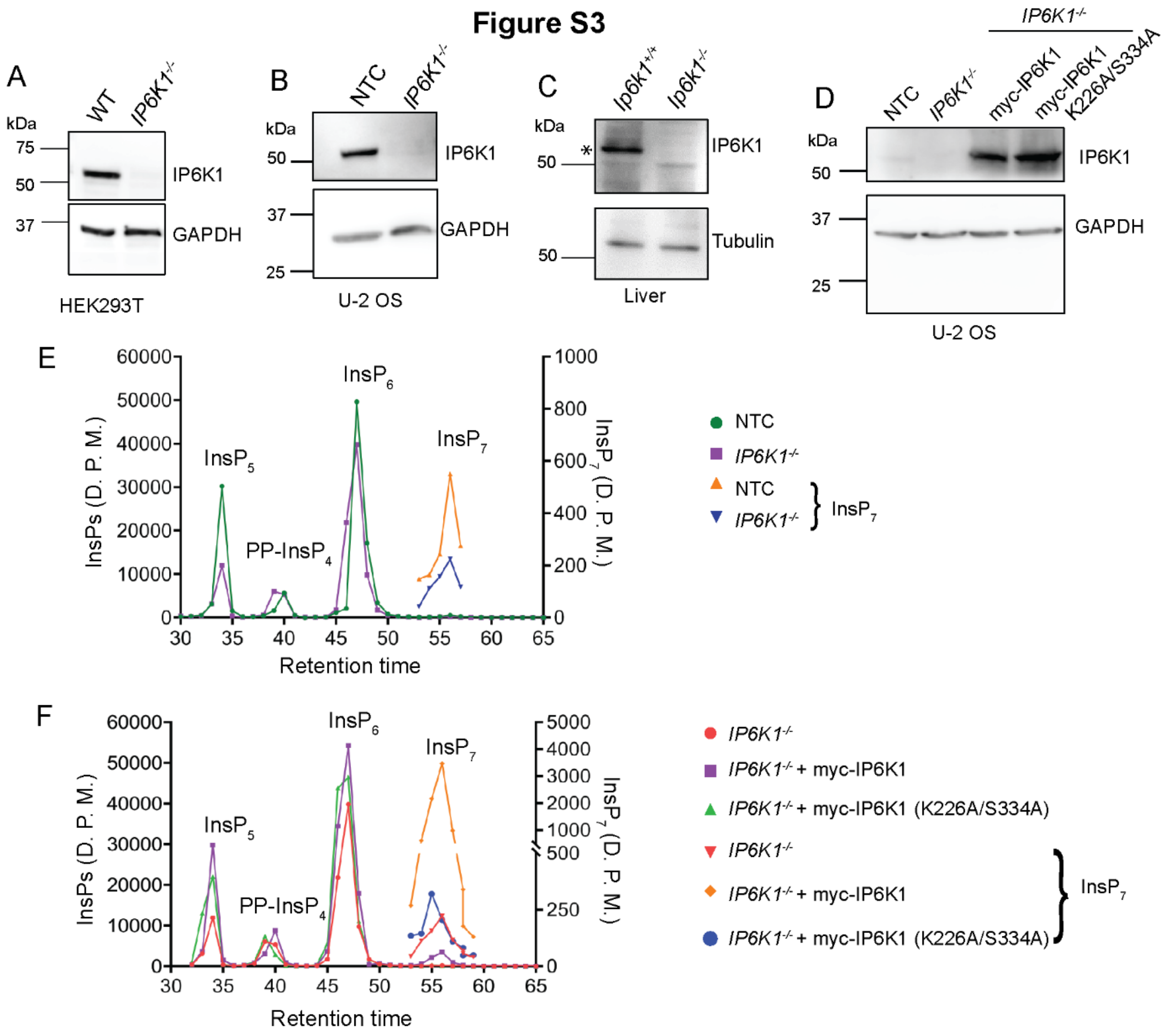

Figure S3. Characterisation of IP6K1 knock out cell lines and mouse liver. (A-C) Representative immunoblots showing the absence of IP6K1 in *IP6K1*$^{-/-}$ knockout HEK293T (A), and U-2 OS (B) cell lines, and *Ip6k1*$^{-/-}$ mouse liver compared with their respective controls (C). **(D)** Representative immunoblot demonstrating the stable expression of myc-tagged active or catalytically inactive (K226A/S334A) mouse IP6K1 in *IP6K1*$^{-/-}$ U-2 OS cell line. **(E-F)** HPLC profiles of [^{3}H]-inositol-labelled NTC and *IP6K1*$^{-/-}$ U-2 OS cells (E), and *IP6K1*$^{-/-}$ U-2 OS cells stably expressing myc-tagged active or catalytically inactive (K226A/S334A) mouse IP6K1 (F). The profile for $InsP_7$ is shown separately (right Y axis), in addition to all InsPs (left Y axis).

Figure S4

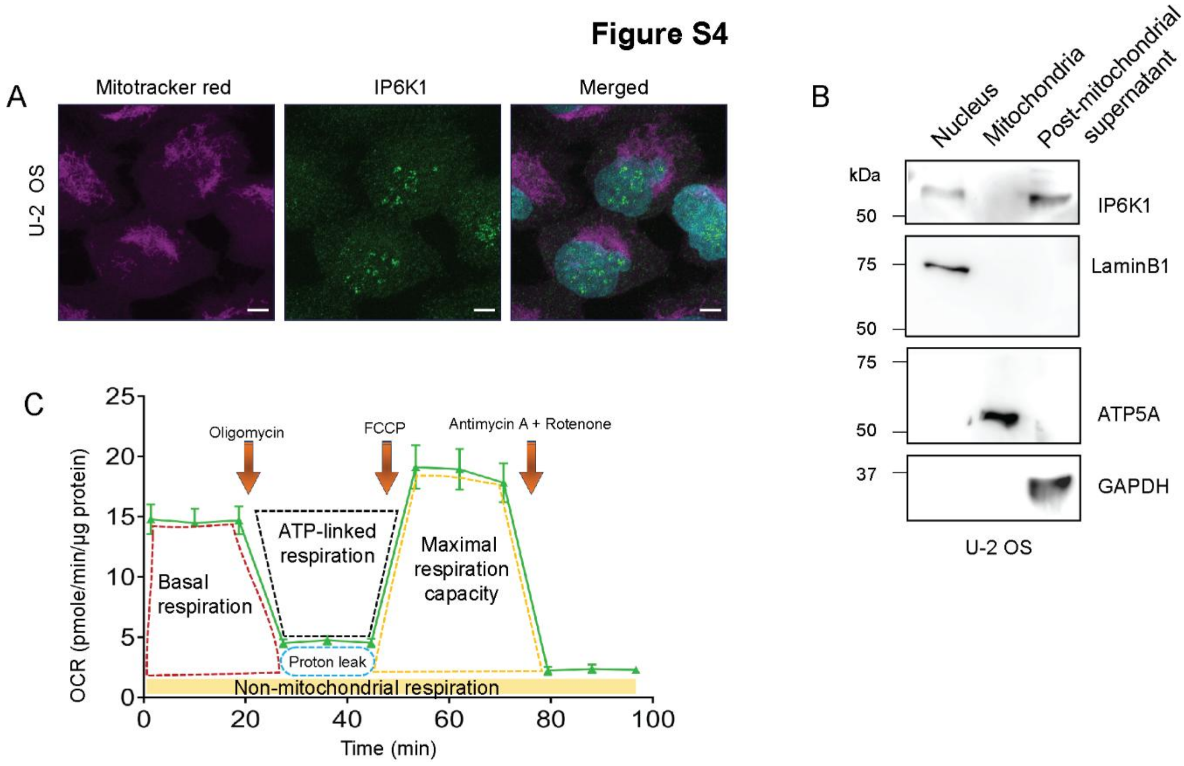

Figure S4. IP6K1 does not localise to mitochondria. (A) Asynchronous U-2 OS cells were stained with Mitotracker Red (Magenta) and anti-IP6K1 antibody (green): scale bars, 5 µm. Co-localization of Mitotracker Red with IP6K1 was assessed by calculating Pearson's correlation coefficient, which yielded a value of 0.069 ± 0.056 (mean ± S.E.M.), indicating no significant co-localisation. **(B)** Representative immunoblot showing the enrichment of IP6K1 in the nuclear and post-mitochondrial supernatant fractions, with no detectable signal in the mitochondrial fraction. **(C)** Schematic representation of the interpretation of OCR traces, expressed as pmoles O_2/min/µg protein. Arrows indicate the time points at which Oligomycin, FCCP, and Antimycin A/Rotenone are added. Baseline cellular OCR is measured initially, from which basal respiration is calculated by subtracting non-mitochondrial respiration. Upon addition of oligomycin (Complex V inhibitor), the decrease in OCR is used to determine ATP-linked respiration (by subtracting the oligomycin-inhibited rate from the baseline OCR). Proton leak respiration is calculated by subtracting non-mitochondrial respiration from the oligomycin-inhibited OCR. Next, FCCP (a protonophore), is added to collapse the inner mitochondrial membrane gradient, allowing the ETC to function at its maximal rate. Maximal respiratory capacity is calculated by subtracting non-mitochondrial respiration from the FCCP-stimulated OCR. Lastly, Antimycin A and Rotenone, inhibitors of complex III and I respectively, are added to shut down ETC function, revealing the non-mitochondrial respiration.
